# Supplementary material for: Exploring the Impact of Pitch-Coated Pottery on Wine Composition: Metabolomics Characterization of an Ancient Technique
Source: Foods. 2025 Nov 11;14(22):3857. doi: 10.3390/foods14223857 (PMC12651153; doi:10.3390/foods14223857)
Supplement: Supplementary file 1 [file foods-14-03857-s001.zip › Supplementary material S1_Foods.pdf]

Exploring the Influence of Pitched Pottery on Wine Production: Using omic techniques for characterization of Traditional Techniques

Abarca-Rivas, Clara[1,2], Lozano-Castellón, Julián[1,2], Vallverdú-Queralt, Anna[1,2], Pérez, Maria[1,2], Corrado, Marina[1,2], Zifferero, Andrea[3], Chessa, Riccardo[4], Rosellini, Daniele[5], Reynolds Paul[6], Lamuela-Raventós, Rosa M.[1,2], Pecci, Alessandra[7]*

Supplementary Material S1

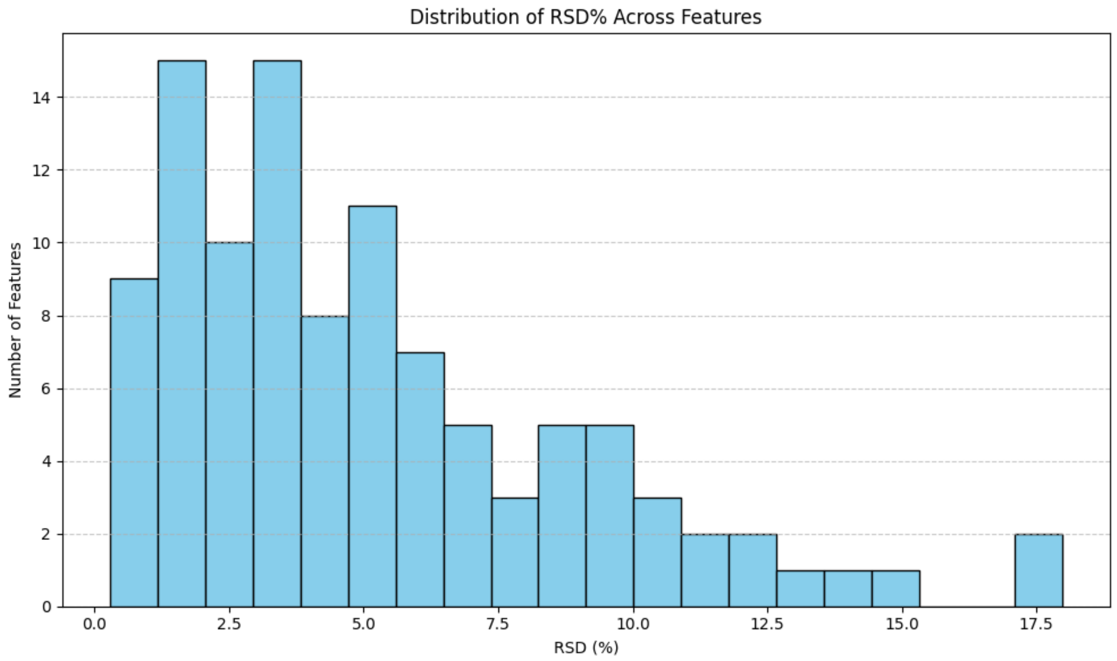

Figure S1. Relative standard deviation (RSD%) of detected features in QC samples

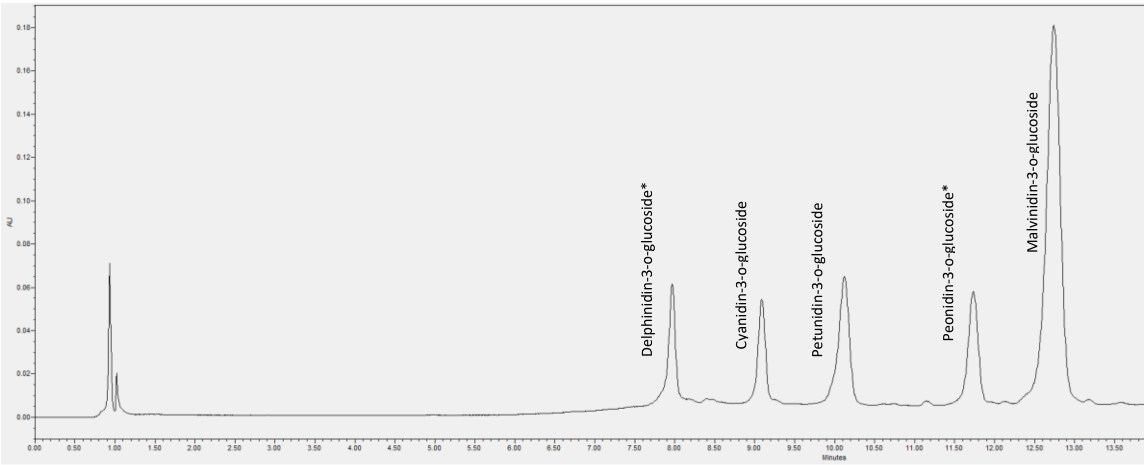

Figure S2. Chromatogram of anthocyanidins reported by UPLC-DAD in experimental wines. Compounds marked with an asterisk were identified by LC-HRMS. The rest were identified by pure standards.

Hotelling’s T2

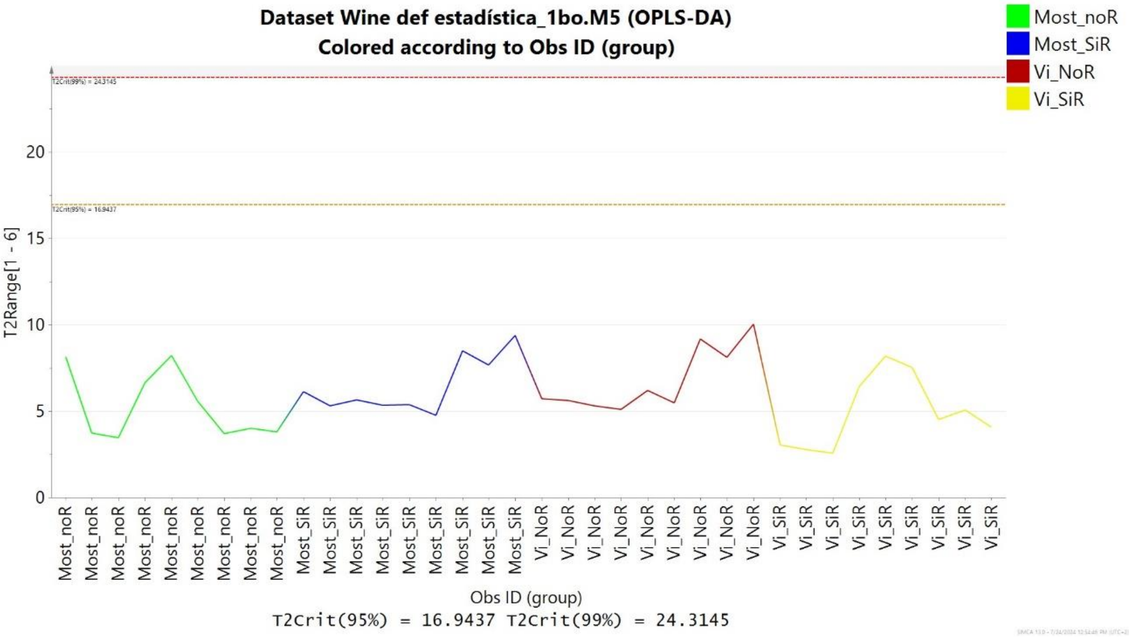

Residuals N-plot

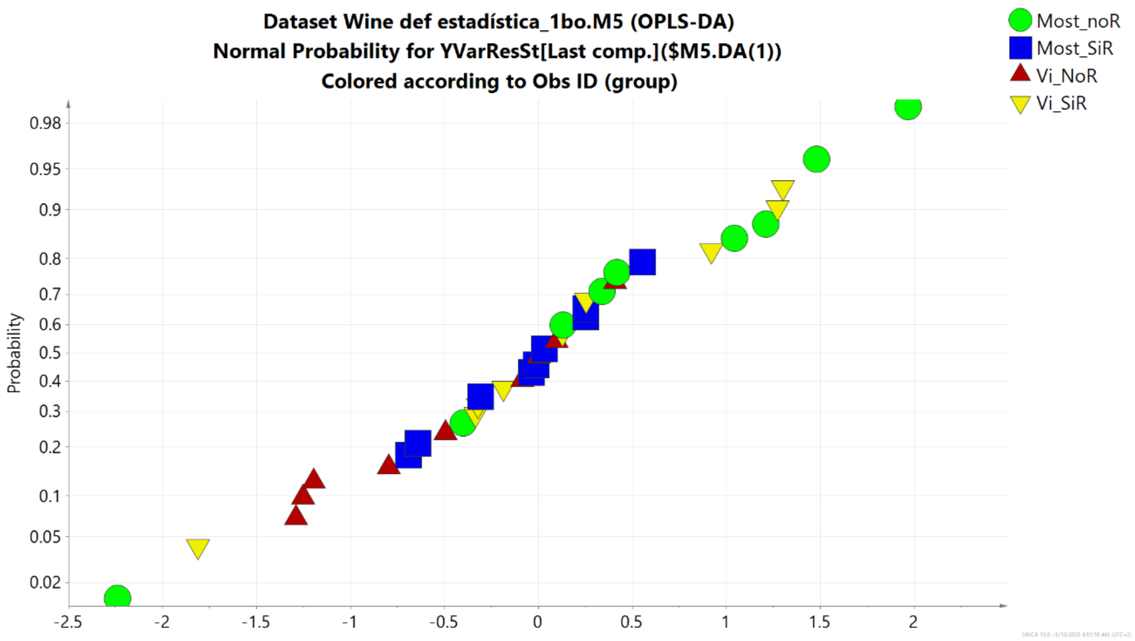

Permutation Plot

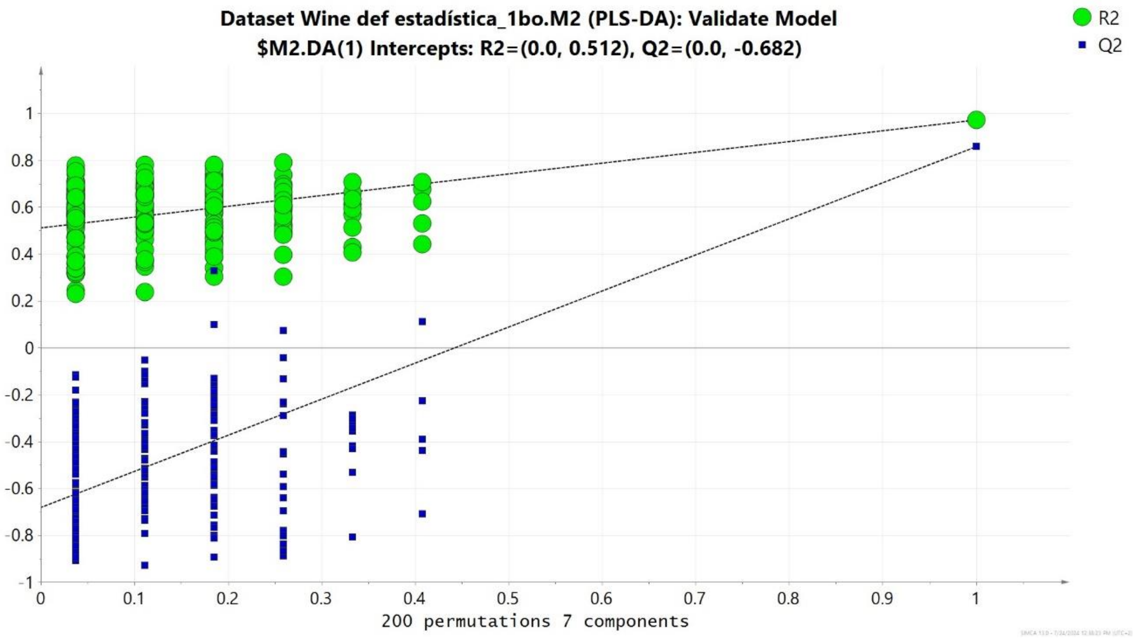

Figure S3. Hotelling's T2, residuals normal probability plot and permutation plots.
